# Environmentally Friendly and All-Dry Hydrophobic Patterning of Graphene Oxide for Fog Harvesting

**DOI:** 10.1021/acsomega.3c06197

**Published:** 2024-02-14

**Authors:** Kurtuluş Yılmaz, Mehmet Gürsoy, Mustafa Karaman

**Affiliations:** Chemical Engineering Department, Konya Technical University, Konya 42030, Turkey

## Abstract

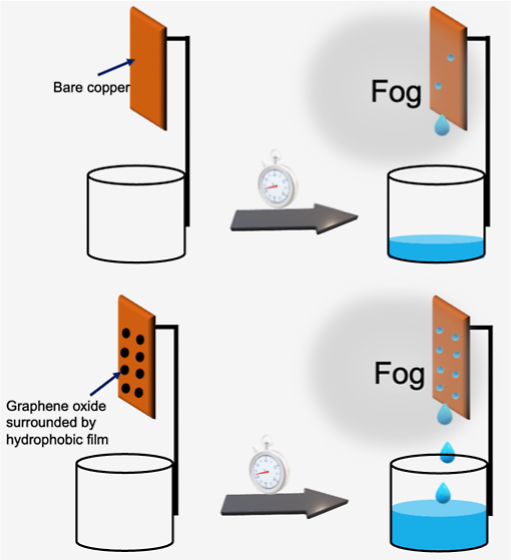

This study examines the fog-harvesting ability of graphene oxide surfaces patterned by hydrophobic domains. The samples were prepared from graphene deposited using low pressure chemical vapor deposition, which was later plasma oxidized to obtain hydrophilic graphene oxide (GO) surfaces. Hydrophobic domains on GO surfaces were formed by initiated CVD (iCVD) of a low-surface-energy poly(perfluorodecyl alkylate) (PPFDA) polymer. Hence, patterned surfaces with hydrophobic/hydrophilic contrast were produced with ease in an all-dry manner. The structures of the as-deposited graphene and PPFDA films were characterized using Raman and Fourier transform infrared spectrophotometer analyses, respectively. The fog harvesting performance of the samples was measured using the fog generated by a nebulizer, in which the average diameter of the fog droplets is comparable to meteorological fog. According to the fog harvesting experiment results, 100 cm^2^ of the as-patterned surface can collect fog up to 2.5 L in 10 h in a foggy environment. Hence, hydrophilic/hydrophobic patterned surfaces in this study can be considered as promising fog harvesting materials. Both CVD techniques used in the production of hydrophilic/hydrophobic patterned surfaces can be easily applied to the production of large-scale materials.

## 1 Introduction

With the intensification of the effects of global warming in many countries around the world, access to fresh water is becoming a big problem threatening the societies. According to a recent report, nearly one-fifth of the world’s population are living in areas with intense water-stress, which is projected to be much worse in the upcoming decades if no action is taken.^1^ Water plays a vital and indispensable role in the lives of communities, affecting nearly everything related to life, such as drinking water, agriculture, industry, hygiene, health, and and so forth. Considering the worst scenario of losing nearly 40% of rain and snow in the upcoming decades, immediate actions must be taken to preserve the societies.^2^ One of the mostly used traditional techniques to obtain clean water is the desalination process of seawater,^3,4^ but the process is highly energy incentive, which will definitely contribute to the global warming through increased carbon emissions to the atmosphere. The disposal of byproduct brine is another big challenge to solve.^5^ Therefore, the need for low-cost and environmentally friendly techniques is gaining importance to reduce the gap between water supply and demand of societies. In recent decades, fog harvesting has become a potential way to produce water in regions with high water stress.^6,7^ This method is mostly suitable for areas with high humidity and are prone to fog formation.

The passive fog-collecting materials in the industrial fog collectors are meshes made from hydrophobic polymers such as positron emission tomography, polypropylene, and nylon.^8^ These materials allow water droplets to form on their outer surfaces through condensation. The as-condensed droplets, which are able to grow in size and merge with others, are collected in containers placed below the meshes. Recently, a biomimetic approach has become a hot topic in fog harvesting studies in order to develop materials or surfaces with maximized fog-collection efficiency.^9−12^ The most recent studies carried out to improve the fog collecting efficiency were based on the combination of hydrophobic and hydrophilic domains on a single surface, which was inspired by Namib desert beetle.^13−15^ Namib desert beetle is a well-known example of natural fog harvesting species, which captures water because of its dorsal surface possessing specially distributed hydrophobic and hydrophilic parts.

In this study, a simple all-dry production scheme is demonstrated in order to prepare a hydrophilic-patterned hydrophobic surface, which showed improved fog-harvesting properties. Graphene oxide (GO) was chosen as the hydrophilic coating, which was produced by plasma oxidation of graphene deposited on a copper substrate by a low-pressure chemical vapor deposition (LPCVD) technique. GO is hydrophilic in nature^16,17^ and thanks to its extremely high thermal conductivity on metallic Cu surface, it does not put a barrier against the heat dissipation of the surface, which helps to keep the surface cool for the enhanced water condensation. As a second step, a thin (<100 nm) film of a low-surface energy fluorinated polymer was deposited on top of hydrophilic GO surface by the initiated chemical vapor deposition (iCVD) method. iCVD is a solvent-free and low-temperature vapor deposition strategy which is suitable to functionalize fragile surfaces with thin polymeric coatings at high levels of functional group retention.^18−20^ In iCVD, the chemical activation of the precursor vapors is achieved by using thin heated wires suspended a few centimeters above the substrate surface. The usage of initiator during iCVD provides depositions at low substrate temperatures.^21−23^ In iCVD, due to the lack of a solvent which would be needed in a classical wet coating strategy, there is no restriction on the compatibility between the surface and the solvent.^24−26^ Hence, a highly hydrophobic material can be placed on a highly hydrophilic material like GO without any restrictions, which otherwise is impossible to do using wet approaches due to the wetting restrictions. Within the scope of this study, spherical particles were placed on top of the GO surface during the iCVD coating, which allowed patterning the surfaces with hydrophilic domains. During the deposition of low-surface energy polymer, the regions of the GO surface in contact with the spherical particles remain uncoated, hence leaving cylindrical trenches of the hydrophilic regions on the surface. The conformal coating ability of iCVD allowed this simple patterning strategy. The diameter of the trenches and the distance between them could be easily adjusted by the selection of spherical particles with different diameters. Hence, a patterned surface with hydrophobic/hydrophilic contrast was produced with ease, and the effect of various parameters on the fog collection ability of the as-produced materials was investigated.

## 2 Experimental Section

### 2.1 Synthesis of Graphene Oxide on Copper Surface

**Figure 1:**
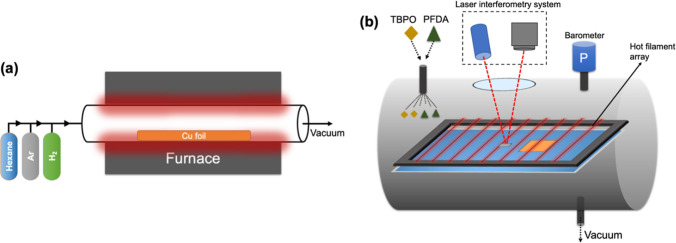
Schematic drawing of (a) LPCVD and (b) iCVD.

Graphene was synthesized by the LPCVD method on a 25 μm thick copper (Cu) foil (Alfa-Aesar, 99.8% purity) surface. Details of the LPCVD system used in this study are given elsewhere.^27^ The schematic drawing of LPCVD is given in Figure 1a. Prior to the deposition, Cu foil was first pretreated with 1 M nitric acid solution to remove the natural oxides from the surface. After the acid treatment, the Cu foil was rinsed in 2-propanol for 5 min and dried with nitrogen gas. The as-treated copper foil was then annealed at 950 °C for 20 min with the hydrogen/argon gas mixture to reduce the surface roughness. The annealing process under hydrogen flow can reduce the roughness of the surface down to 0.41 nm root mean square, as shown in our previous publication.^27^ In that way, an atomically clean and flat Cu surface can be obtained, which is suitable for the growth of high-quality graphene. After these initial surface preparation steps, LPCVD of graphene was started through exposing the as-treated Cu foil to a mixture of 27 sccm Ar and 2 sccm hexane (Sigma-Aldrich, 97%) gases at a reactor temperature of 900 °C. After a fixed deposition duration of 15 min, the graphene-deposited Cu foil was removed from the LPCVD chamber and transferred to an oxygen plasma cleaner (Diener, Femto, Germany) for plasma oxidation of the graphene surface. Inside the plasma cleaner, the samples were exposed to oxygen plasma for 10 s under a plasma power of 100 W.

### 2.2 Hydrophobic Modification of GO Surface by iCVD Method

Thin films of PPFDA were deposited on the surface of GO by the iCVD method to make the surface hydrophobic. The details of the iCVD system used in this study are given elsewhere.^28^ The schematic drawing of iCVD is given in Figure 1b. During iCVD, perfluorodecyl acrylate (PFDA) was used as the monomer and ditertbutyl peroxide (TBPO) was used as the initiator. PFDA and TBPO were vaporized in separate stainless-steel jars which were connected to the iCVD reactor by 6 mm-diameter stainless steel piping. The flow rates of the vaporized precursors were controlled by needle valves placed on the connection pipes. In the iCVD system, the substrate to be coated was placed on the cooling stage, the temperature of which was kept constant at 32.5 °C using water from a recirculating chiller. The energy required for activation of the precursors was provided from a nichrome (Ni–Cr 80/20 wt %, 0.3 mm diameter) filament array, which was placed 2.5 cm above substrate. During the depositions, the filament temperature was kept constant at 240 °C. The reactor pressure was kept constant at 500 mTorr, which was measured by a capacitance type pressure sensor (MKS Baratron).

**Figure 2:**
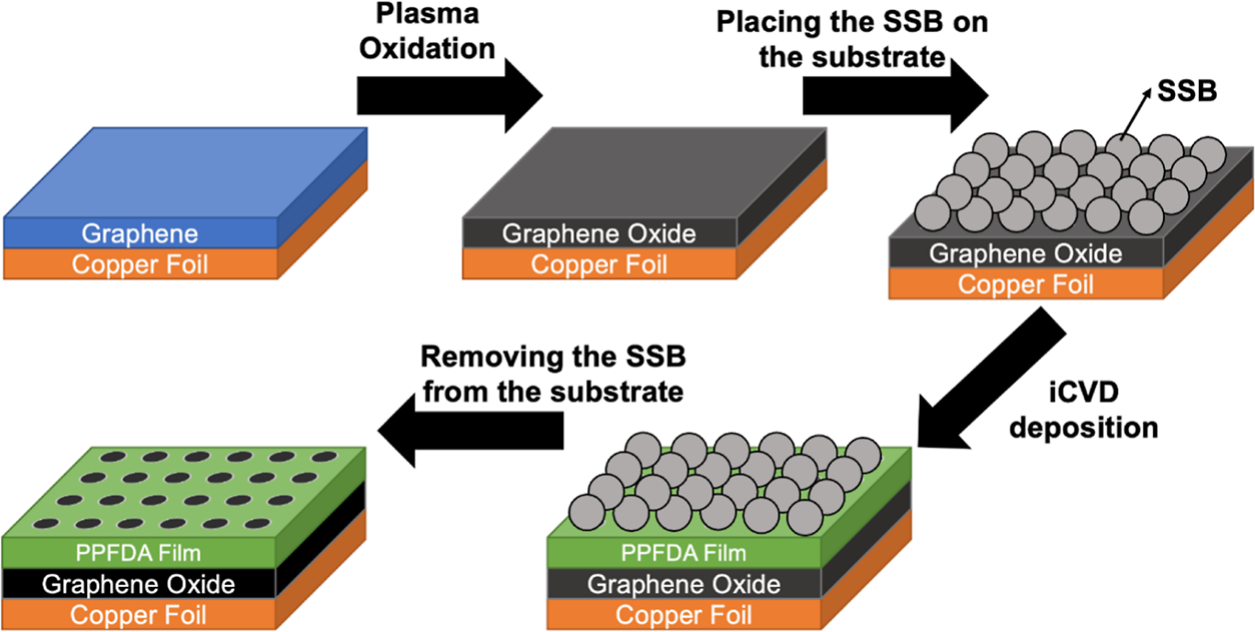
Schematic representation of fabrication process for hydrophilic GO/hydrophobic PPFDA patterned surface on copper.

The patterning method applied during iCVD is schematically (not to scale) shown in Figure 2. In order to make hierarchically patterned trenches of GO domains on the surface, arrays of spherical stainless-steel balls (SSBs) were used for masking the as-grown graphene oxide film surface. The diameter of the trenches and the distance between them could easily be adjusted by using SSBs having different diameters, namely, 2 and 4 mm. The samples produced by using 2 and 4 mm SSBs in the patterning are named PS 1 and PS 2, respectively. Before iCVD, SSBs were directly placed on top of the GO sheet, which was previously mounted on the cooling plate of the iCVD chamber. SSBs had enough weight to keep them fixed on the substrate surface during iCVD so that no extra fixing on the surface was required. To further prevent the unexpected rolling of the SSBs during iCVD, a vacuum was created slowly by using a throttle valve between the chamber and the pump. After the iCVD deposition of PPFDA, balls were removed from the surface. In that way, the contact points of the SSBs remained uncoated and hydrophilic, while the other places of the surface were coated uniformly with PPFDA film.

### 2.3 Materials Characterization and Fog Harvesting Measurements

The quality of graphene deposited from LPCVD before and after plasma oxidation was analyzed by Raman spectroscopy (inVia, Reinshaw). Raman spectra were obtained using a confocal Raman spectrophotometer at an excitation wavelength of 532 nm. The structure of the iCVD PPFDA film was analyzed with a Fourier transform infrared spectrophotometer (FTIR, Thermo Scientific, Nicolet IS10) using an attenuated total reflectance accessory.

FTIR spectra were obtained in the range of 500–4000 cm^–1^ wavenumbers at a resolution of 4 cm^–1^ averaged over 32 scans. The changes in water contact angle values (WCA) of surfaces before and after different surface-modification stages were revealed using a contact angle goniometer (Kruss Easy Drop). Image recording settings were adjusted to 17.4 s at 62 FPS, and the analysis method was based on the Young–Laplace equation. For each WCA measurement, 4.0 μL pure water droplets with pH value close to 7 was placed on the surface. In addition to static WCA measurements, advancing and receding WCA measurements of hydrophobic samples were performed. Advancing and receding WCAs were measured by increasing and decreasing the droplet volume, respectively, until the contact line was observed to move.^29^ The WCA measurements were repeated at least three times for each sample. AFM (NT-MDT) was applied to measure the average roughness (*R*_a_) and root-mean-square (*R*_q_) values of the samples under semicontact mode with scan areas of 5 × 5 μm^2^.

The fog harvesting performance of the samples (25 mm width and 50 mm length) was measured using a custom-made fog generation (Figure S1). In this setup, fog was generated by an ultrasonic humidifier (PulseMed, Model: GL-2205), which generates humid air with water droplets 0.5–6.0 μm in diameter. The fog was directed to the samples through a pipe with a fan at the outlet of the nebulizer. The air temperature and the relative humidity near the samples were measured as 25.1 ± 0.5 °C and 90–99%. The samples were mounted over a beaker placed on an electronic mass balance. The distance between the sample and the humidifier nozzle was 10 cm. The fog harvesting measurements were repeated three times for each sample.

## 3 Results and Discussion

### 3.1 Sample Characterization

**Figure 3:**
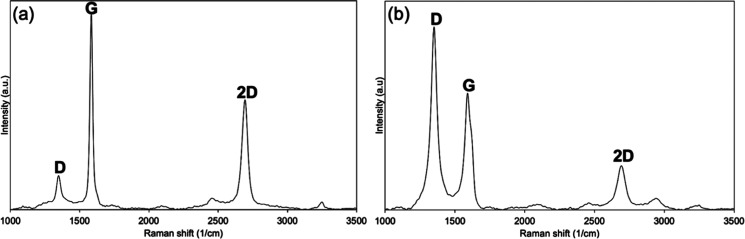
Raman spectra of (a) graphene and (b) graphene oxide.

Figure 3a,b shows the Raman spectra of LPCVD graphene deposited on the Cu substrate before and after plasma oxidation, respectively. The Raman spectrum of as-deposited graphene possesses two peaks which are known as the fingerprint bands for graphene: G band appearing at around 1580 cm^–1^ and 2D band appearing at around 2700 cm^–1^. The shape, position, and relative intensity of the G and 2D Raman peaks depend mostly on the number of graphene layers.^30^ In this study, the relative intensities of 2D and G bands, namely, 2D/G peak intensity ratio, were used to assess the number of layers of the as-deposited graphene. From Figure 3a, the intensity ratio was calculated as 0.67, which implies the deposition of few-layer graphene. Apart from that, the D-band observed around 1350 cm^–1^ represent the presence of structural defects resulted from the broken sp^2^ bonds. The ratio of intensity of D/G bands is a good measure of the level of disorder and defects present on the graphene structure.^31,32^ D/G peak intensity ratio was very low at 0.17 before the plasma exposure but significantly increased to 1.6 after oxygen plasma exposure, which can be associated with an increase in the defect density due to the defects resulting from the broken sp^2^ bonds during harsh plasma conditions.

**Figure 4:**
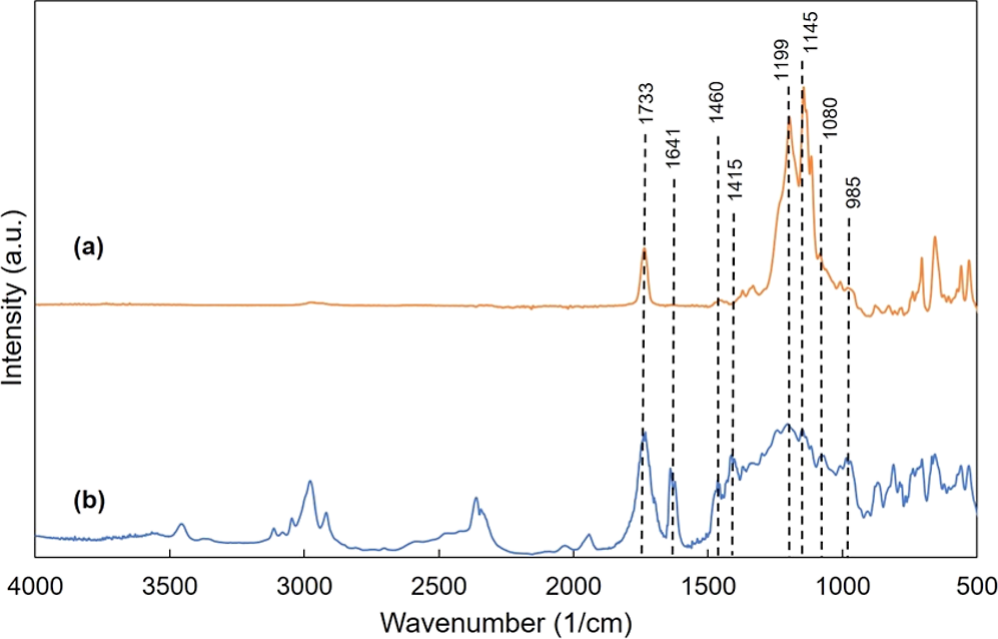
FTIR spectra of (a) iCVD PPFDA and (b) liquid monomer.

The PPFDA film deposited on the GO surface was characterized using FTIR. Figure 4 compares the FTIR spectrum of PPFDA (top) and the PFDA monomer (bottom). The peak observed at 1733 cm^–1^ in both spectra is due to the C=O stretching. In the PPFDA spectrum, the intense peak at 1145 cm^–1^ is caused by the –CF_2_–CF_3_ end group. The other sharp peaks observed at 1080 and 1199 cm^–1^ are caused by the asymmetric stretching and symmetric stretching of the –CF_2_–, respectively.^33−37^ The monomer spectrum contains peaks originating from the C=C double bond at 1641, 1460, 1415, 1080, and 985 cm^–1^, which are absent in the polymer spectrum indicating that the polymerization pathway during iCVD is through the double bond of the vinyl monomer.^36^ Hence, it can be concluded that iCVD is able to produce PPFDA films on GO with high retention of the pendant perfluoroalkyl group of the monomer.

**Figure 5:**
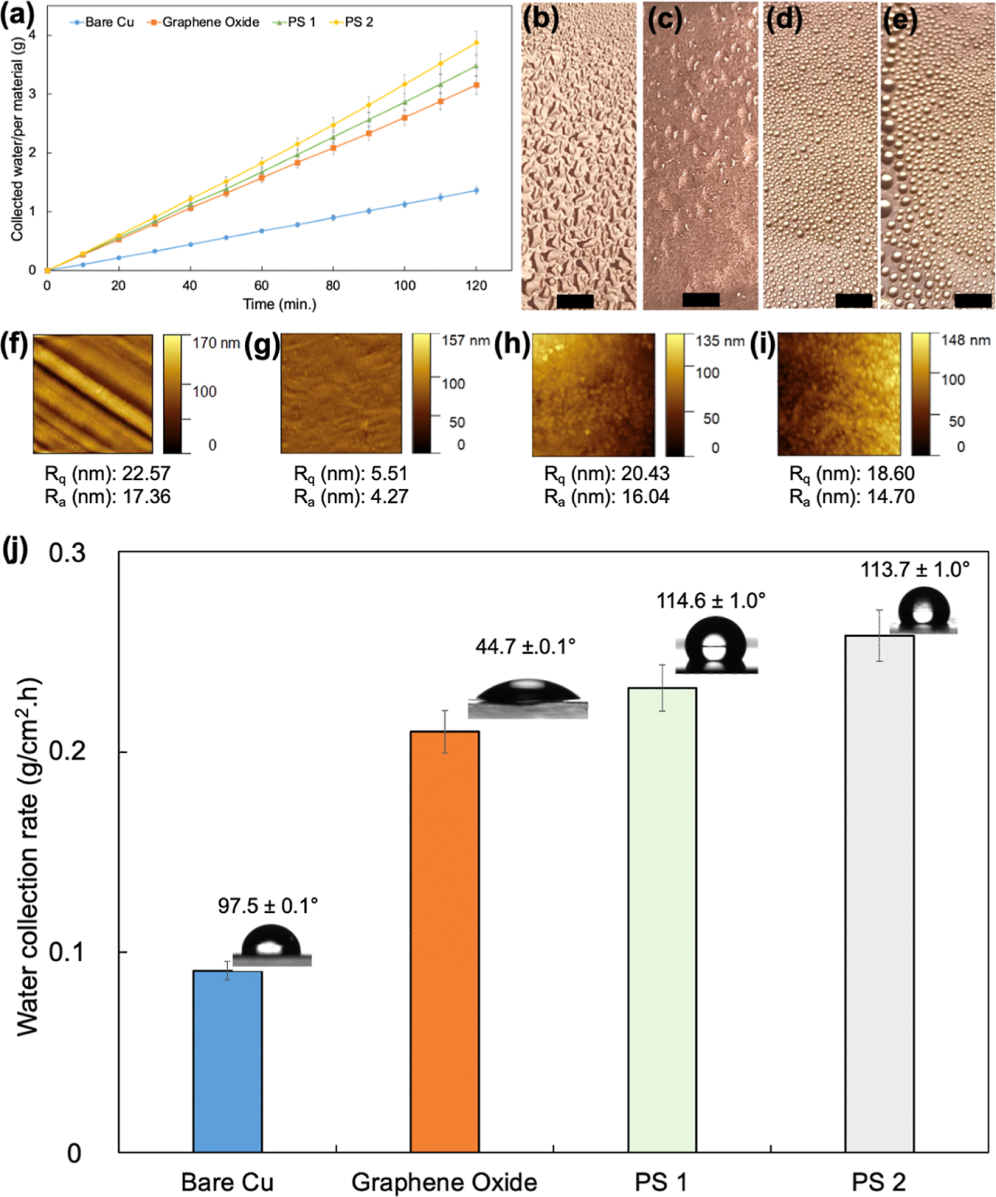
(a) Fog harvesting onto different materials over time, the photograph of (b) bare Cu, (c) graphene oxide, (d) PS 1, and (e) PS 2 exposed to fog. (Scale bar, 0.2 cm), AFM images with the roughness values of (f) bare Cu, (g) graphene oxide, (h) PS 1, (i) PS 2, and (j) water collection rate (contact angle values are written in the relevant materials).

Because the surface wettability significantly influences the fog collection, the surface wettabilities of as-prepared surfaces at different stages of the synthesis scheme are characterized by measuring static WCA values. The wetting characteristics of samples are given in Figure 5j. The untreated copper surface is slightly hydrophobic with a WCA of 97.5 ± 0.1°. After graphene deposition, the WCA value of the surface decreased to 89.5 ± 0.1°. This value is in line with the reported contact angle values of graphene deposited on copper surface by the LPCVD technique.^38^ The number of graphene layers has a direct effect on the measured WCA values on any surface on which graphene is deposited. It was observed in literature that as the number of graphene layers increases, the contact angle difference between graphene deposited and uncoated surface increases.^39^ From the Raman analysis, the as-deposited graphene had a multilayer, which explains the difference between the measured WCA values. The plasma surface oxidation of the graphene resulted in a dramatic decrease in WCA value due to the formation of hydrophilic oxide moieties on the surface after plasma treatment. The deposition of PPFDA by iCVD method turned the surface highly hydrophobic with measured WCA values of 113.7 ± 1.0° and 114.6 ± 1.0°. The difference between the measured WCA values originated from the hydrophilic GO domains created using spherical SSBs with different diameters. The deposition of the same PPFDA film on the reference Si wafer surface resulted in a WCA value of 120.5 ± 0.5°. Indeed, the maximum static WCA observable on a flat surface is typically around 120°, since greater WCA values can be achieved only on rough surfaces.^40^ The decrease in the overall WCA value on the PPFDA-coated surface is due to the existence of the hydrophilic GO domains. The contact angle hysteresis values of PS1 and PS2 were calculated as 41.8 ± 2.0 (advancing WCA: 122.1 ± 1.2, receding WCA: 80.3 ± 2.9) and 40.8 ± 2.8 (advancing WCA: 119.8 ± 2.2, receding WCA: 78.9 ± 1.6) respectively. It is not surprising that PS1 and PS2 samples, which have a flat surface structure with hydrophilic regions on their surfaces, show relatively high contact angle hysteresis values. Furthermore, in order to prove the stability of the coatings, WCA measurements were obtained before and after the fog harvesting experiments, and it was observed that there is not a significantly important difference in the WCA values. This observation indicates that the coatings remained stable on the surface after the fog harvesting experiments.

### 3.2 Fog-Harvesting

The fog harvesting performances of hydrophilic, hydrophobic, and hydrophilic/hydrophobic patterned surfaces were compared, as shown in Figure 5a. Bare copper and graphene oxide were used as hydrophobic and hydrophilic materials, respectively. An important reason for the use of graphene oxide as a hydrophilic material in this study is that besides its hydrophilic nature, it is durable and insoluble in water.^41^ When polar water molecules reach the hydrophilic surface, hydrogen bonds are expected to form between the surface and the water.^42^ On hydrophilic surfaces, the adhesion, spreading, and condensation of fog droplets to the surface can easily take place, which is essential for efficient and sustainable fog harvesting.^43^ Therefore, we can expect the graphene oxide surface to collect many more fog droplets. This could be the reason why the GO coated copper surface collected showed better fog harvesting performance as compared with that of the bare copper surface. Actually, for an effective fog harvesting, not only water absorption ability but also low re-evaporation rate of the collected water and easy separation of the water from the surface are also important.^44^ Hydrophobic surfaces allow faster removal of droplets deposited on the surface.^45,46^ While water absorption is related to hydrophilicity, evaporation rate and separation of water from the surface are also related to hydrophobicity.^47^ Looking at the photographs of the surfaces exposed to fog (Figure 5b–e), when comparing bare copper with graphene oxide, it can be suggested that the bare copper surface is not hydrophobic enough to allow water to flow. Besides the hydrophilicity and hydrophobicity of the surface, there are other important factors affecting the fog harvesting performance of a material, such as the surface morphology and chemical structure of the materials or the conditions of the environment (flow velocity and incidence angle of water droplets from the nebulizer, the distance between the nebulizer and the material, etc.). Therefore, hybrid wettability surfaces can be expected to exhibit better fog harvesting performance compared to uniform wetting (either hydrophilic or hydrophobic) surfaces.^48^

Since hydrophilic/hydrophobic patterned surfaces have the advantages of both wettability properties, they can be considered as an ideal surface for fog harvesting. When creatures with the ability to harvest fog in nature are investigated, it has been observed that their binary or different degrees of wettability patterned surface structures play an important role in this ability.^49,50^ Many researchers have developed fog harvesting materials inspired by such natural structures.^51−53^ Studies showed that binary wettability patterned materials harvest more fog droplets than materials with uniform wettability, either hydrophilic or hydrophobic.^54^ Surfaces with hydrophilic regions surrounded by a hydrophobic background are one of the most effective fog harvesting patterns.^55,56^ The fog collection mechanism on such surfaces is based on the accumulation of fog drops in hydrophilic regions until they reach a certain weight and the flow of the droplets leaving this region through the hydrophobic area. The main motivation in the patterning method performed here was to obtain such a patterned surface. As seen in Figure 5d,e, the droplets collected on the patterned surfaces did not spread like those on the other surfaces. As it reached a certain weight, the drops eventually fell due to gravity to the beaker. AFM images of the samples with *R*_a_ and *R*_q_ values were presented in Figure 5f–i. According to the AFM results, it was observed that the roughness of the sample surfaces increased with the PPFDA thin film coating. The most important reason for this may be that the long perfluoroalkyl groups of PFDA have natural tendencies to reorient themselves to form large crystal structures.^57^ In our study, it is very difficult to claim that there is a direct relationship between surface roughness and fog harvesting performances. It can be suggested that surface chemistry plays a more dominant role in the fog harvesting efficiency. However, it should be noted that the contact angle value increases with increasing surface roughness. This observation is in agreement with Wenzel and Cassie–Baxter models showing the relationship between surface roughness and contact angle in hydrophobic materials.^58,59^

Based on the World Health Organization report, for a single individual, the minimum water requirement to sustain life under moderate climatic is about 2.5 L per day.^60^ In this study, the size of the fog droplets generated by the nebulizer is comparable with meteorological fog.^61^ Both CVD techniques (thermal CVD and iCVD) used here in the production of hydrophilic/hydrophobic patterned surfaces can be easily applied to the production of large-scale materials. According to the fog harvesting experiment results from Figure 5j, 100 cm^2^ PS2 produced in this study when exposed to fog for 10 h can collect more than 2.5 L in a foggy environment. Therefore, hydrophilic/hydrophobic patterned surfaces in this study can be considered as promising fog harvesting materials.

## 4 Conclusions

In this study, an environmentally friendly and all-dry method was developed to fabricate graphene oxide surfaces patterned with hydrophobic thin films for efficient fog harvesting. Among surfaces exposed to artificial fog with diameters similar to meteorological fog, it was observed that graphene oxide materials with hydrophobic patterns harvested more fog than materials with uniform wettability, either hydrophilic or hydrophobic. Considering the scale-up potential of both LPCVD and iCVD techniques used in the study, it is considered possible to produce large scale fog harvesting materials with the method developed here for real-world applications. According to the results, if 100 cm^2^ of hydrophobic patterned graphene oxide surfaces are produced and exposed to fog for 10 h, it can meet the daily fresh water needs of one person. The approach developed in this study can be applied to fabricate various patterned graphene surface for other applications such as sensors and devices.

## Supporting Information Available

Figure of the fog harvesting setup (PDF)
