# Supplementary material for: Environmentally Friendly and All-Dry Hydrophobic Patterning of Graphene Oxide for Fog Harvesting
Source: ACS Omega. 2024 Feb 14;9(8):8810–7. doi: 10.1021/acsomega.3c06197 (PMC10905578; doi:10.1021/acsomega.3c06197)
Supplement: Supplementary file 1 — ao3c06197_si_001.pdf [file ao3c06197_si_001.pdf]

Environmentally Friendly and All-dry Hydrophobic Patterning of Graphene Oxide for Fog Harvesting

(Supporting Information)

Kurtuluş Yılmaz[a], Mehmet Gürsoy [a,1*], Mustafa Karaman[a,1*]

[a] Chemical Engineering Department, Konya Technical University, Konya, 42030, Turkey

*Corresponding Authors:

E-mail: mgursoy@ktun.edu.tr, mkaraman@ktun.edu.tr

Phone: +(90) 332 223 1972, +(90) 332 223 2108

Fax: +(90) 332 241 0635

Postal address: Department of Chemical Engineering, Konya Technical University, Campus, Konya 42030, Turkey

[1] These corresponding authors have made equal contributions.

1. Figures

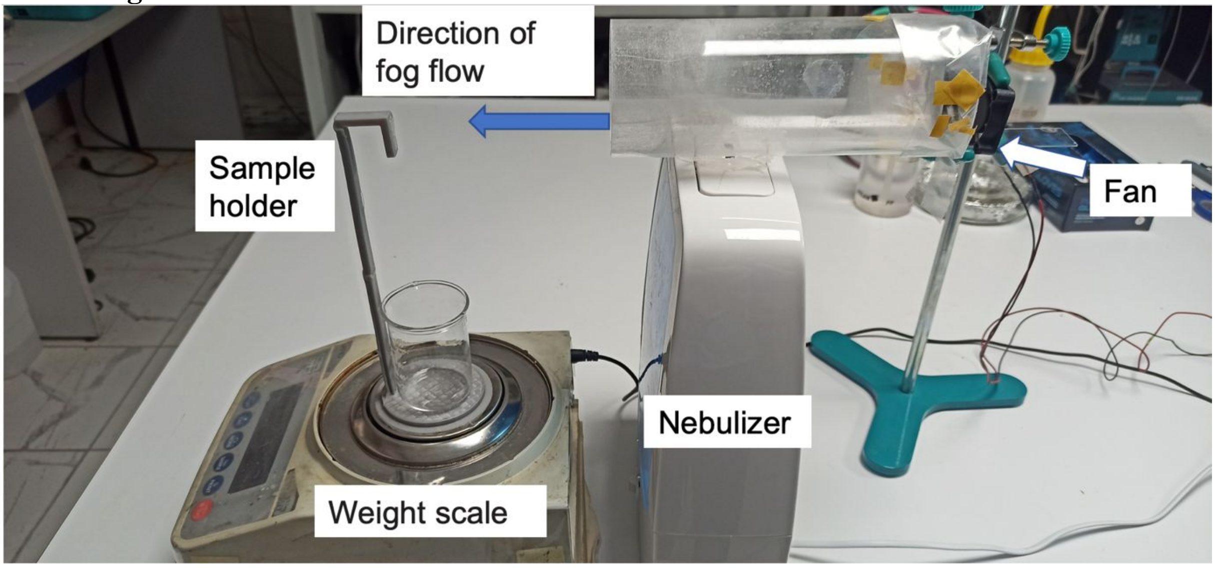

Figure S1. Fog harvesting set-up
